# Antitumor Activity of Tenacissoside H on Esophageal Cancer through Arresting Cell Cycle and Regulating PI3K/Akt-NF-*κ*B Transduction Cascade

**DOI:** 10.1155/2015/464937

**Published:** 2015-09-30

**Authors:** Yong-sen Jia, Xue-qin Hu, Hegyi Gabriella, Li-juan Qin, Nora Meggyeshazi

**Affiliations:** ^1^College of Chinese Medicine, North China University of Science and Technology, Tangshan 063000, China; ^2^Institute of Orthopaedics and Traumatology, Zhejiang Chinese Medical University, Hangzhou 310053, China; ^3^Health Science Department, Pecs University, Pecs 7622, Hungary; ^4^School of Basic Medicine, North China University of Science and Technology, Tangshan 063000, China; ^5^First Department of Pathology & Experimental Cancer Research, Semmelweis University, Budapest 1085, Hungary

## Abstract

*Objective*. The purpose of the study was to elucidate the molecular mechanism of tenacissoside H (TDH) inhibiting esophageal carcinoma infiltration and proliferation. *Methods*. *In vitro*, EC9706 cells were treated with TDH. Cells proliferation and cell cycle were assayed. PI3K and NF-*κ*B mRNAs expression were determined by real time PCR. *In vivo*, model of nude mice with tumor was established. Mice were treated with TDH. Inhibition ratio of tumor volume was calculated. PCNA expression was examined. Protein expression in PI3K/Akt-NF-*κ*B signaling pathway was determined. *Results*. *In vitro*, TDH significantly inhibited cells proliferation in a time-and-dose-dependent manner. TDH arrested the cell cycle in S phase and significantly inhibited PI3K and NF-*κ*B mRNA expression, compared with blank controlled group (*P* < 0.05). In vivo, TDH strongly inhibits tumor growth and volume. PCNA expression was significantly decreased after treatment of TDH. TDH downregulated proteins expression in PI3K/Akt-NF-*κ*B transduction cascade (*P* < 0.05). *Conclusion*. TDH inhibited esophageal carcinoma infiltration and proliferation both *in vitro* and *in vivo*. The anticancer activity has relation to arresting the cell cycle at the S phase, inhibited the PCNA expression of transplanted tumors in nude mice, and regulated the protein expression in the PI3K/Akt-NF-*κ*B transduction cascade.

## 1. Introduction

Incidence and mortality of esophageal cancer (EC) in China ranks the 1st around the world, particularly, in rural areas; for example, at the border region of Henan, Shanxi, and Hebei Province of China [1], average incidences reach 46.64/100,000 and mortality reaches 36.95/100,000 [2]. The reason for high incidence of EC in china is malnutrition and absence of trace elements for peoples in some areas. In regions around Taihang Mountain, huge numbers of citizens develop a habit of eating sauerkraut; food nitrosamines and mold food may be the leading factor of EC in these areas. Furthermore, inadequate nutrition and lack of molybdenum in soil may be cancer-promoting factors [3]. The Linxian General Population Nutrition Intervention Trial (NIT) was carried out during 1985–1991, and the findings indicated that a combination of beta-carotene, vitamin E, and selenium may result in a reduction in esophagus risk in this population [4]. The results of this study provided some evidence of malnutrition for EC etiology.

As an important complementary and alternative medicine, traditional natural medicinal herbs show great predominance at treating the disease in recent years, characterized with significant effect and few side effects. Bioactive substances in herbs have recently aroused the interest of scientists for their potential use in prevention and treatment of many diseases, including cancer.

Tenacissoside H (molecular formula: C_42_H_66_O_14_, TDH) is a Chinese medicine monomer extracted. It is isolated from* Caulis Marsdeniae Tenacissimae*, which is also named “Black bone vine,” being a precious Chinese medicinal plant that grew in the Southwest Yunnan-Guizhou Plateau region [5]. The medical value of* Caulis Marsdeniae Tenacissimae* has been known for thousands of years. It is widely and traditionally used for its anti-inflammatory effects. The plant contains novel anticancer substances which are mainly the C21-steroidal glycosides and total alkaloids that could eliminate cancer hormones and disrupt cancer cell proliferation or metastasis [6].

This present study was carried out to explore antitumor activity of TDH on human esophageal carcinoma. The results showed that TDH inhibited EC development* in vitro* and* in vivo*. Concrete mechanism related to arresting EC9706 cell cycle at the S phase inhibits proliferating cell nuclear antigen (PCNA) of transplanted tumors in nude mice and regulates PI3K/Akt-NF-*κ*B transduction cascade.

## 2. Materials and Methods

### 2.1. Cell Culture

Human esophageal carcinoma EC9706 cells were donated by Tumor Institute, Beijing Union Medical College. EC9706 cells are squamous carcinoma cells in esophagus, which are the chief type of EC in China, accounting for more than 95% in whole EC. So if EC9706 cells may be influenced by tenacissoside, it is very helpful to elucidate anticancer activity of tenacissoside.

EC9706 cells were incubated in RPMI1640 culture medium supplemented with 10% new-born calf serum (CS), penicillin (100 U·mL^−1^), and streptomycin (100 *μ*g·mL^−1^). Cells were maintained at 37°C in a humidified incubator with 5% CO_2_ and regularly examined with an inverted microscope. Media were changed every two days and cells were subcultured at 70–80% confluence.

### 2.2. Drugs and Reagents

Tenacissoside H (TDH) was obtained from National Institutes for food and Drug Control (lot: 111913-201402). 5-Fluorouracil injection (5-FU, as a positive controlled drug, PCD) was produced by Tianjin Jinyao Amino Acid Co., Ltd. (lot: H12020959). RPMI1640 culture medium and new-born calf serum (CS) were purchased from GiBCO Company of America. Enzyme trypsin was from Cusabio Company in China. Methylthiazolyldiphenyl-tetrazolium bromide (MTT) and dimethyl sulfoxide (DMSO) were from Amresco Company of America. Ribonuclease (RNase) A and propidium iodide (PI) were from Sigma-Aldrich Company of America (lots G75485 and H95632). The following 6 antibodies were purchased from CST Company of America, including EGFR monoclone antibody, PI3K polyclone antibody, Akt monoclone antibody, phospho-Akt monoclone antibody, NF-*κ*B polyclone antibody, and PCNA monoclone antibody. The cDNA synthesis and fluorescent quantitative PCR kits were provided by ABI Ambion of America, and PI3K and NF-*κ*B upstream and downstream primers were synthesized by Generay Biotechnology Co. Ltd. of China.

### 2.3. Instruments and Equipment

Laminar flow cabinet was from FORMA Company (1100, America). Ultra sonication instrument was purchased from SONIC Company (VCX750, Japan). CO_2_ incubator was purchased from FORMA Company (3336, America). Inverted microscope was provided by NIKON Company (TMD-2, Japan). Fluorescence microscope was purchased from NIKON Company (EF-D, Japan). High speed centrifuges of low temperature were purchased from Eppendorf Company and SIGMA Company (R134A and 3MK, America). Electrophoresis meter was purchased from ATTO Company (AE-6531, mPAGE, Japan). EIA photometer was purchased from Bio-Tek Company (NOVAPATH 450, America). Real-time PCR was purchased from ROCHE Company (LightCycler 480, Germany).

### 2.4. Cell Proliferation Assay

EC9706 cells were seeded in 96-well plates at a concentration of 1 × 10^4^ cells/well with complete culture medium and allowed to adhere to the plate for 24 h. The adherent cells were incubated in the presence of various concentrations of TDH (0, 2, 4, 6, 8, and 10 mg·mL^−1^) for another 24 h and 48 h; MTT assay was conducted. The Optical Density (OD) absorbance of the treated samples against Blank Controlled (BC) Group was measured with EIA photometer with the wavelength of 570 nm; the same experiment was repeated 3 times, according to the following formula [7]:

(1) Inhibition  rate  of  EC9706  cells  regulated  by  TDH=1−OD  of  treated  groupsOD  of  controlled  group×100%.

Fitting curve of dose-effect dependence was protracted and median effective dose (IC50) was worked out by SPSS18.0 software.

### 2.5. Cell Cycle Analysis

Cells were seeded on *Ø*100 mm plates at a concentration of 1 × 10^6^ cells/dish, 10 mL culture medium/dish, and incubated at 37°C in a 5% CO_2_ incubator for 24 h. TDH (10 mg·mL^−1^) and PCD (5 FU, 10 mg·mL^−1^) added in RPMI1640 culture medium were present for another 24 h. 3 mL of trypsin was applied into dishes; cells were harvested into 15 mL centrifuge tubes individually. The collected cells were centrifuged at 1000 rpm for 10 min, and, after 2 mL PBS was applied, the cells were centrifuged at 1000 rpm/min for 5 min, 2 times. A total of 100 *μ*L PBS and 70% ethanol were applied into dishes and then left at 4°C overnight. After the cells were fixed at 4°C, 3 mL/tube of cold PBS was added, resting for 1 min, and then centrifuged at 1000 rpm for 5 min again. PBS was abandoned; then a mixture of solution, containing 850 *μ*L of PBS, 10 *μ*L of RNase A, 100 *μ*L of 1% Triton-100, and 40 *μ*L of 1 mg/mL PI, was added; cells were incubated for 5 min. Cells were then filtered through 400-mesh screen, cell cycle was determined by flow cytometry, and the result was analyzed by Modifit LT software [8].

### 2.6. RT Fluorescence Quantitative PCR Assay

EC9706 cell culture condition was the same as the above mentioned in Cell Cycle Analysis. Four time points were set for sampling after different concentration of compound treatment, including day 1, day 3, day 5, and day 7. Total RNA was extracted from the cells; 4 *μ*L of total RNA was mixed with random primers, reverse transcriptase enzymes, and other reactants to a final volume of 20 *μ*L system. RNA was reverse transcribed to cDNA at 42°C for 15 min, at 95°C for 2 min, and processed for RT fluorescent PCR detection. PCR conditions were 94°C 30 s, 1 cycle, and 95°C 5 s, 60°C 35 s, total 40 cycles. Relative concentrations of mRNA were calculated by comparing with the Ct value of Group BC at day 1. Primers sequences of PI3K, NF-*κ*B p65, and *β*-actin are as in Table 1.

### 2.7. Mice Modeled with EC9706 Cells and Administered with Drugs

All mice were maintained in individual ventilation cages, constant humidity being 50–80%, relative temperature keeping 18–22°C, and luminosity for 10 hours. The mice were fed with standard fodder for nude mice and water freely. This animal protocol was approved by the Animal Care Welfare Committee of North China University of Science and Technology. Female and male Balb/c nude mice aged six weeks, weighing between 18 and 22 g, were obtained from National Institutes for Food and Drug Control (China), number for animal certificate of approval being SCXK (Beijing) 2009-0017. Animal Center of North China University of Science and Technology meets requirement for nude mice experiment. The protocol lasted for 24 days, including adaptive feeding for 3 days, 7 days for tumor growth, and 14 days for treatment.

EC9706 cells were cultivated in normal condition; 1 × 10^7^/mm^3^ of the exponential phase of EC9706 cells was transplanted and inoculated subcutaneously into the right flank of nude mice, 1.5 mL/20 g body weight. The tumor growth was monitored and measured three times a week with a vernier calliper. After 7 days from cells being transplanted, tumors were 100–300 mm^3^ in size, and then mice were randomized into 3 groups. Each of TDH and PCD group consisted of 6 animals. There were 12 animals in the negative model group (Group NM). TDH and 5 FU were dissolved with saline separately. 100 mg·kg^−1^ of TDH was injected intraperitoneally twice a week and 20 mg·kg^−1^ of 5-FU twice a week. Mice in Group NM were treated at the same time with 20 mL·kg^−1^ of saline.

### 2.8. Tumor Xenograft Study

All mice were observed for 14 days from the beginning of treatment. The tumor volume (*V*) was calculated by the following formula:

(2) $$\begin{array}{c} V\left(\;{\text{mm}}^{3}\;\right)=\frac{\text{length}\times {\text{width}}^{2}}{2}. \end{array}$$

See [9].

Relative tumor volume (RTV) is calculated by dividing the tumor volume at any time by the tumor volume at the beginning of treatment. The tumor growth inhibition index (*T*/*C*%) was calculated according to the following formula:

(3) $$\begin{array}{c} \frac{T}{C}\left(\;\%\;\right)=\frac{T_{RTV}}{C_{RTV}}\times 100\%, \end{array}$$

where *T*_RTV_ is relative tumor volume of the treated group. *C*_RTV_ is relative tumor volume of the control group [10].

After 14 days, all mice were sacrificed through cervical dislocation. The tumors were harvested for immunohistochemical analysis and real-time fluorescence quantitative PCR.

### 2.9. Immunohistochemical Staining

Tumor tissues were collected, excised, and fixed in 4% paraformaldehyde, embedded in paraffin, and sectioned. Sections were preheated, dewaxed, gradient dehydrated in alcohol, oxidized in 3% (v/v) hydrogen peroxide at room temperature for 15 min, and rinsed 3 times with 0.01% (v/v) PBS. Sections were then incubated overnight at 4°C with PCNA primary antibody (1 : 100 dilution) in a wet box. Sections were dropped by biotin-labeled secondary antibody and incubated at room temperature for 2 h, detected with diaminobenzidine (DAB) solution containing 0.006% hydrogen peroxide. Hematoxylin was used as a counterstain. After washing with PBS, mounting medium was applied and sections were coverslipped and imaged. Only the first tumor slice from each rat was chosen for analysis. Five randomly selected fields of view were captured under a ×200 optical microscope and the percent of positive cells were counted and averaged.

### 2.10. Western Blot

Supernatant of tumor tissue homogenate was collected. Protein concentration was determined with Bradford method [11]. The solution was moved to the centrifuge tube and stored in the −20°C condition. 8% SDS-PAGE separating gel and stacking gel was applied.

Every sample was transferred in gels for electrophoresis. Electrotransfer was conducted for 8 hours. Ponceau was used for dying. The standard protein marker was labeled. Sealing fluid immerses nitrocellulose filter to block nonspecific proteins. Antigen-antibody reaction lasted for 4 hours. ECL color liquid was added, special protein expression transfers onto the X-rays, and exposure and developed film were followed. Image J software (developed by National Institute of Health of USA) was applied to analyze protein gray value.

### 2.11. Statistical Analysis

Continuous variables were expressed as mean ± standard error. All samples were tested to ascertain if they followed homogeneity of variance by One-Way ANOVA. On the premise of data at normal distribution, least-significance difference (LSD) was applied to compare data difference between every two groups. If data were at abnormal distribution, Dunnett's T3 was used. A value of *P* < 0.05 was regarded as statistically significant. SPSS 18.0 statistical software (SPSS, Chicago, IL, USA) was used for statistical analysis.

## 3. Results

### 3.1. MTT Assay for Drugs Sensitivity to EC9706 Cells* In Vitro*

**Figure 1:**
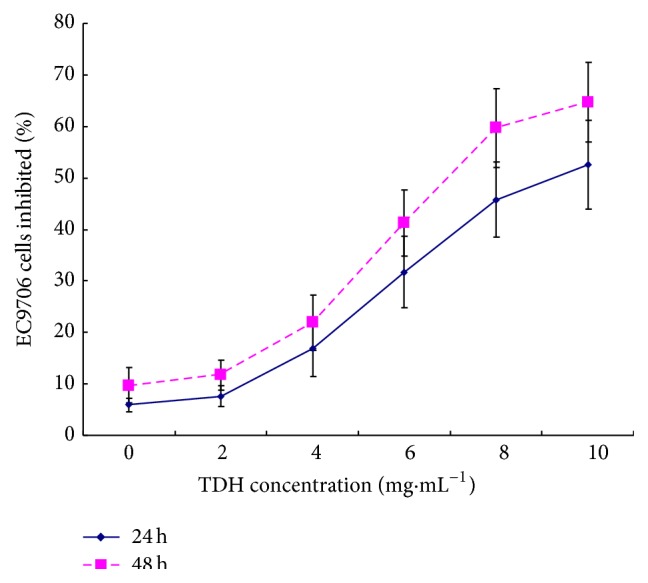
Percentage of cell proliferation rate. TDH inhibited EC9706 cells proliferation in a dose- and time-dependent manner. Human esophageal cancer EC9706 cells were seeded in 96-well plates and treated with various concentrations of TDH for 24 and 48 h; TDH was diluted in the RPMI1640 medium. Data were expressed as mean ± standard error. Six samples were tested on each treatment. The experiment was repeated three times.

MTT assay was used to investigate the growth inhibitory effect of TDH on human esophageal cancer EC9706 cells. Cells were exposed to various concentrations of TDH for 24 and 48 h. Result showed that TDH has a stronger inhibitory effect on EC9706 cells than the control group. The inhibition rates of TDH were 52.65% and 64.79% at 10 mg·mL^−1^ for 24 and 48 h, respectively. These results showed that TDH significantly inhibited cells in a time- and dose-dependent manner (Figure 1). 50% growth inhibition concentration (IC50) of TDH was (9.81 ± 1.57) and (6.45 ± 1.68) mg·mL^−1^ for 24 and 48 h, respectively.

### 3.2. Cell Cycle Assayed by Flow Cytometry

**Figure 2:**
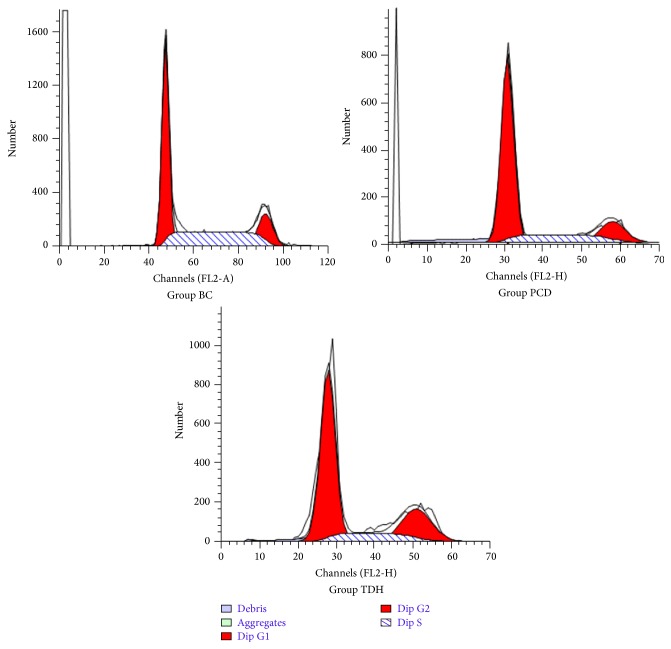
Cell cycle analysis in EC9706 cells. Cells are calculated to 50,000 in each group by a flow cytometry produced by Brady Company, type FACSCalibur FCM. The method is PI dying. Three samples are observed in each group. The dosage of drugs is as follows: in Group BC, RPMI 1640 medium containing 10% CS; in Group PCD, 5 FU concentration 10 mg·mL^−1^ (diluted with RPMI 1640 medium); in Group TDH, TDH concentration 10 mg·mL^−1^ (diluted with RPMI 1640 medium).

**Figure 3:**
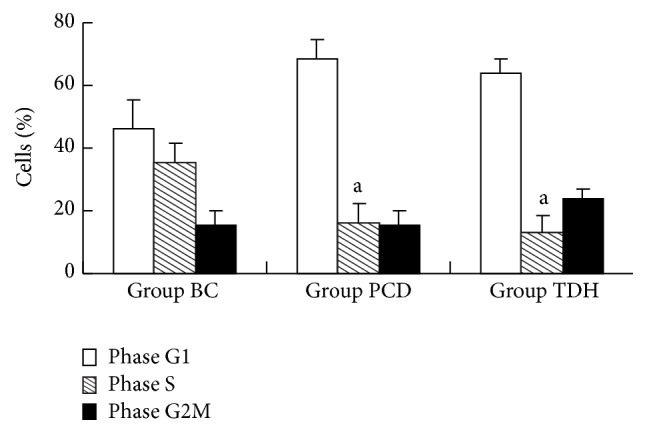
Cell cycle measurement for EC9706 cells regulated by TDH and 5 FU. Data are expressed as mean ± standard error. Data are analyzed by LSD test, ^a^ *P* < 0.05 versus Group BC. Results are considered statistical significance at *P* < 0.05. Three samples are observed in each group. The dosage of drugs is as follows: in Group BC, RPMI 1640 containing 10% CS; in Group PCD, RPMI 1640 containing 5 FU 10 mg·mL^−1^; in Group TDH, RPMI 1640 containing TDH 10 mg·mL^−1^.

Cells percentage in S phase in Group TDH and PCD was much lower than that in Group BC. Percentages of cells in S phase of medicinal groups were (12.82 ± 2.13)% (Group TDH) and (15.26 ± 3.85)% (Group PCD), much higher than that in Group BC (35.59 ± 5.70)% (Figure 2). There was significant difference between medicinal groups and Group BC (*P* < 0.05); however, within the medicinal groups, percentages show no difference (Figure 3).

### 3.3. Expression of PI3K and NF-*κ*B p65 mRNAs in Cells Influenced by Tenacissoside H* In Vitro*

**Figure 4:**
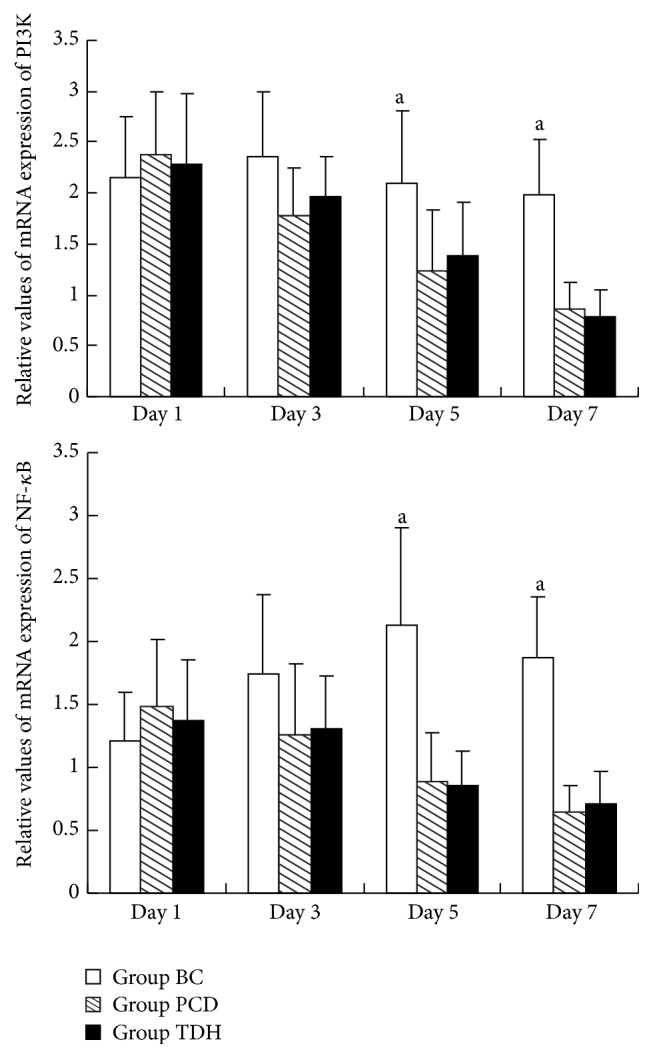
Comparison of PI3K mRNA and NF-*κ*B mRNA expression in cells affected by TDH and 5 FU at different time points RT. The determination method is fluorescence quantitative PCR assay. Manipulation accords to kit's instruction. Data are analyzed by LSD test, ^a^ *P* < 0.05 versus Group BC. Results are considered statistical significance at *P* < 0.05. Three samples are observed in each group. The dosage of drugs is as follows: in Group BC, RPMI 1640 containing 10% CS; in Group PCD, RPMI 1640 containing 5 FU 10 mg·mL^−1^; in Group TDH, RPMI 1640 containing TDH 10 mg·mL^−1^.

Upregulation of PI3K mRNA in Group BC was proportional to time extension and reached the peak at the 3rd day. In contrast, PI3K mRNA expression in Group PCD and TDH showed another tendency. With medication time prolonged, the mRNA took on reduction at different degrees. Peak value of NF-*κ*B p65 mRNA in Group BC appeared at the 5th day. In the medicinal groups, the expression presented decrease from the 3rd day. At the 5th and 7th day PI3K and NF-*κ*B mRNAs concentration in Groups PCD and TDH was statistically significant with that in Group BC (*P* < 0.05), as shown in Figure 4.

### 3.4. Antitumor Effect of Tenacissoside H* In Vivo*

After intraperitoneal injection of each medicine in nude mice, tumor volume was in different degree of inhibition. The tumor volume in Group NM is larger than that in Groups PCD and TDH, showing significant difference (*P* < 0.05). But there was no statistical difference between Groups PCD and TDH, as shown in Table 2.

### 3.5. Histomorphology Change* In Vivo*

**Figure 5:**
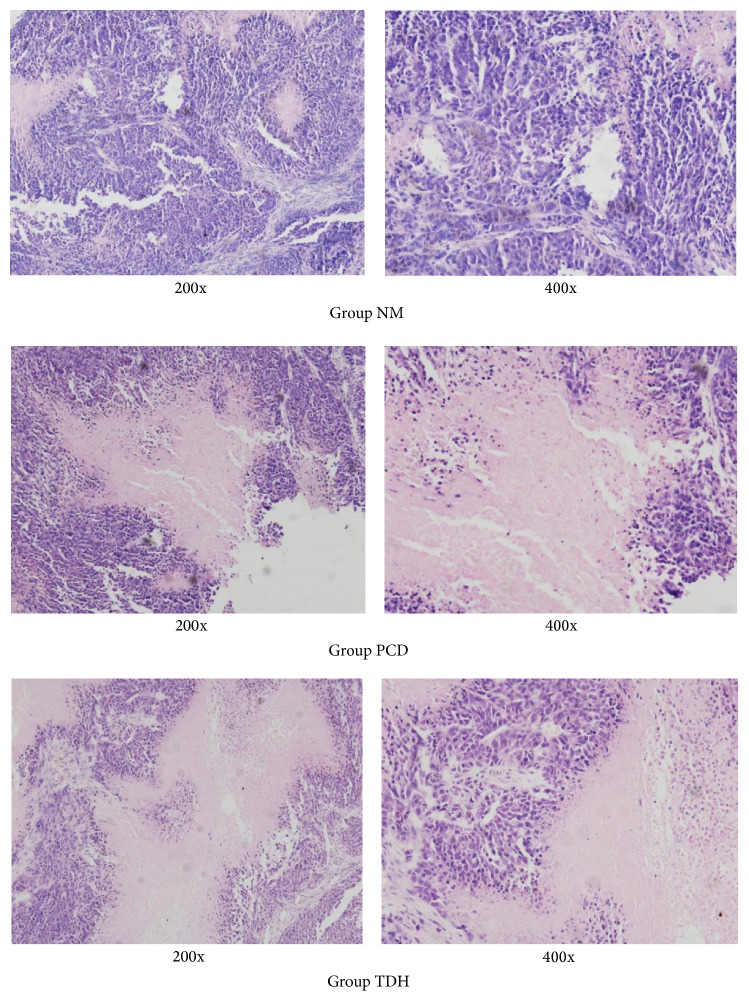
Histomorphology of transplanted tumor was observed with fluorescence microscope. Histomorphology of tumors in different groups is observed with hematoxylin-eosin (HE) staining (200x and 400x). 5 FU and TDH were dissolved by saline to certain concentration. The dosage of drugs is as follows: in Group NM, saline 20 mL·kg^−1^; in Group PCD, 5 FU 20 mg·kg^−1^; in Group TDH, TDH 100 mg·kg^−1^.

Pathological type of tumor tissue in Group NM was characterized with poorly differentiated carcinoma; the nucleolus of tumor cells was rich of glands and blood sinus with hyperchromatism. Tumor tissues in Groups PCD and TDH were light stained, accompanied with large part of necrosis that appeared. There are amorphous materials in necrosis area at high magnification, as shown in Figure 5.

### 3.6. PCNA Change* In Vivo*

**Figure 6:**
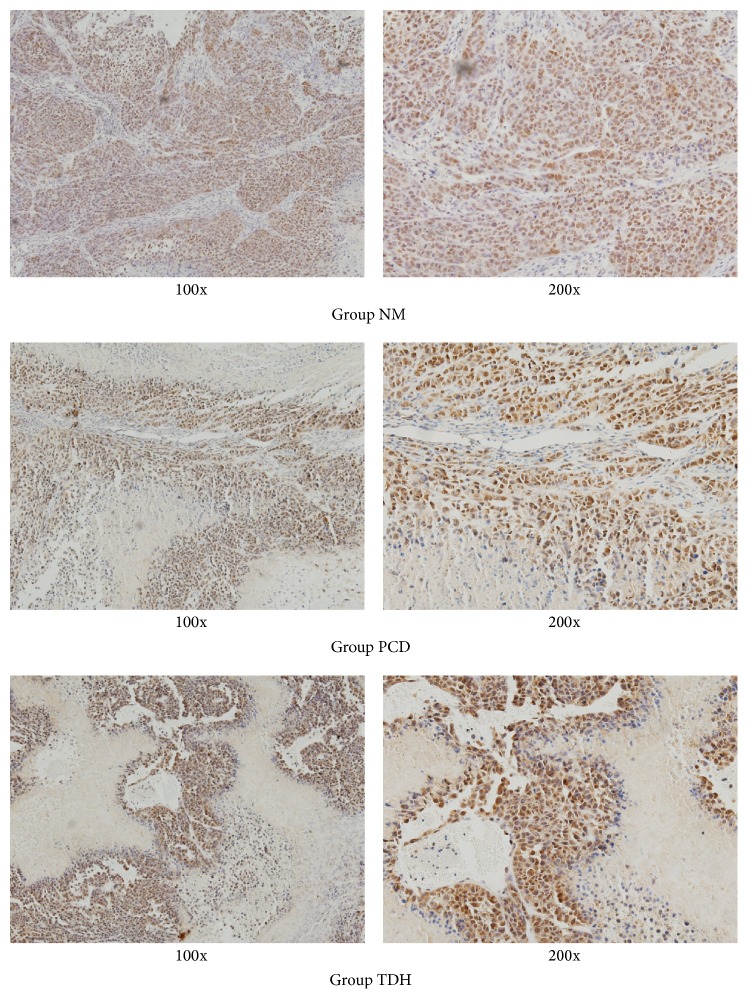
PCNA expression in tumor tissues assayed with immunohistochemical staining. Immunohistochemistry was performed to measure PCNA in tumor tissues derived from control and treated mice (magnification 100x and 200x). 5 FU and TDH were dissolved by saline to certain concentration. The dosage of drugs was as follows: in Group NM, saline 20 mL·kg^−1^; in Group PCD, 5 FU 20 mg·kg^−1^; in Group TDH, TDH 100 mg·kg^−1^. Cells characterized with positive expression of PCNA took on brown. Cells with negative took on blue.

As shown in Figure 6, positive expression of PCNA took on brown, which was very obvious in Group NM, nearly besprinkled in the field of vision. However, negative expression of PCNA, which took on blue, was apparent in Groups TDH and PCD. By semiquantitative analysis in Table 3, expression of PCNA showed significant difference between medicinal groups and Group NM, which indicated that TDH could positively downregulate expression of PCNA, as shown in Figure 6 and Table 3.

### 3.7. PI3K/Akt-NF-*κ*B Signaling Pathway Assayed by Western Blot* In Vivo*

**Figure 7:**
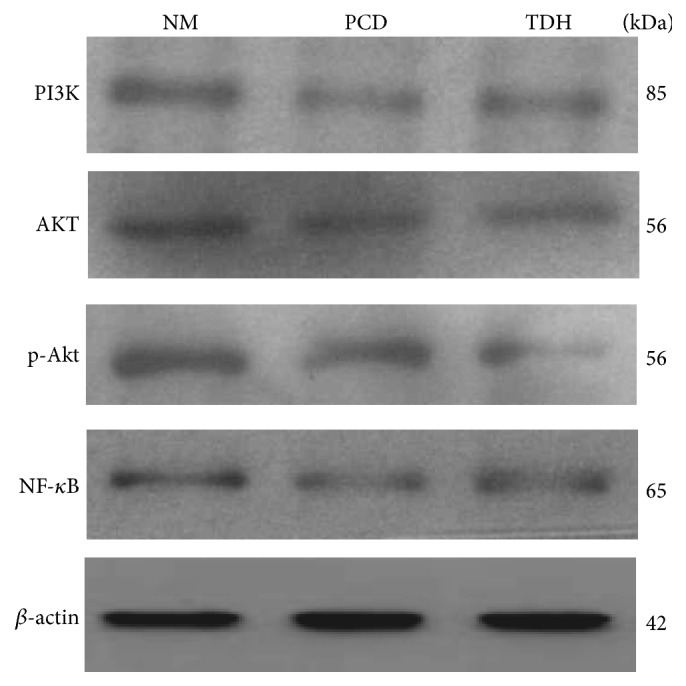
Protein expression in PI3K/Akt signaling pathway regulated with drugs. Protein expression was assayed by western blot. Grey bands appeared to be differently influenced by different drugs. The dosage of drugs was as follows: in Group NM, saline 20 mL·kg^−1^; in Group PCD, 5 FU 20 mg·kg^−1^; in Group TDH, TDH 100 mg·kg^−1^.

The protein expression of PI3K, Akt, p-Akt, and NF-*κ*B was measured by western blot. TDH showed significant inhibition on proteins expression of PI3K/Ak-NF-*κ*B signaling pathway. Compared with Group NM, proteins expression in Group TDH strongly indicates different trend (Figure 7). By semiquantitative analysis with Image J software, gray values of 5 proteins in Group TDH appeared significantly weaker than those in Group NM (*P* < 0.05) (Table 4).

## 4. Discussion

As a very popular medicinal Chinese herb,* Caulis Marsdeniae Tenacissimae* is applied extensively in clinic in China. Its anticancer activity attracts more and more attention among oncologists; the explicit mechanism is explored by professional researchers. But till nowadays, the comprehensive molecular evidences are still not understood. Based on related experiments on it, common digestive cancer in China—esophageal cancer—was used as a target cell line, and the monomer from* Caulis Marsdeniae Tenacissimae*—TDH—was comprehensively investigated overall through serial experiments* in vitro* and* in vivo*.

TDH effectively inhibits the volumes and weights of tumors in nude mice (Table 2); it means that the results from cells proliferation assay are consistent with the cell inhibition rate. Furthermore, HE staining showed (Figure 5) that tumor tissues and cells of nude mice treated with TDH had pale staining; the center of tumor tissue is necrosis or apoptosis, which indicate that TDH significantly inhibits the tumor growth and could lead to necrosis or apoptosis.

Previous related studies had demonstrated that cell growth and proliferation of several kinds of tumor cells depend on cell cycle progression. Induction of cell cycle arrest by a nontoxic phytochemical could be an effective strategy to check the uncontrolled proliferation and the survival of cancer cells. Therefore, the cell cycle distribution was investigated in this study to find out whether TDH can arrest cell cycle at any specific stage in EC9706 cells.

A typical cell cycle has four phases, G1, S, G2, and M, which is controlled by the signal transduction transmembrane and the feedback loop. Abnormal modulation for cell cycle is an important mechanism of carcinogenesis [12]. Malignant tumor cell cycle beyond control for some reason will hinder cells differentiation and proliferate excessively [13]. Cell proportion in phases of S and G2M reflects whether proliferation is active in tumor cells group, which, to some extent, indicates the condition of cell proliferation [14]. Cells in G1 phase always indicate that they are still in depression condition. After TDH was applied, the distribution of cells in the G1, S, and G2M phase changed a lot compared with that in Group BC. As is shown in Figures 2 and 3, cells in S phase were less than those in Group BC after TDH and 5-FU were applied, which showed that TDH inhibited Ec9706 cells proliferation through blocking up cells from entering S phase from G1.

Unlimited proliferation is an important biological characteristic of malignant cancer cells. Carcinoma cells proliferation activity is a good objective index to reflect tumor proliferation potential. As a tumor marker, PCNA is a kind of cell cycle protein, found in the nucleus, which plays an important role in the synthesis of DNA. PCNA is not expressed at the very early stage of G0, begins to increase in G1 phase instead, and reaches peak in S phase; lastly the expression decreased obviously in G2/M phase. If cancer cells proliferate actively, especially cells in S phase characterized with DNA synthesis, the expression of PCNA will increase significantly. For this reason, PCNA is regarded as an index to assess state of cell proliferation and is always used to judge the malignant degree and prognosis of cancer [15]. The results showed that the positive expression rate of PCNA in Group NM was close to 100%, which corroborated EC9706 cells proliferation actively. As shown in Figure 6 and Table 3, PCNA positive expressions nearly balanced between Groups TDH and PCD, both of which showed significant difference from that in Group NM (*P* < 0.05). The results can be used to explain that TDH has good stability of downregulation on PCNA expression of EC cells to effectively regulate cell cycle.

The PI3K/Akt growth signaling pathway comprises a series of serine/threonine kinase cascades that regulates a variety of cellular processes including cell cycle progression, cell survival and migration, and protein synthesis. Alterations in the PI3K/Akt signaling pathway have been implicated in the occurrence and development of human cancer [16]. Activation of the PI3K/Akt pathway has been demonstrated to promote survival of esophageal cancer cells* in vitro,* as well as tumorigenicity and metastasis of human esophageal cancer* in vivo* [17, 18].

The phosphorylation of Akt (p-Akt) further activates NF-*κ*B [19] cascade. A specific function of NF-*κ*B is to promote cell survival through the induction of target genes. Normal and cancer cells apoptosis can be inhibited by the product of NF-*κ*B. Once NF-*κ*B is activated, it can activate the downstream molecules such as TNF-alpha and IL-1*β*, so as to promote tumor cell proliferation, invasion, and metastasis. This experiment focused the effects of TDH on PI3K/Akt and NF-*κ*B signaling pathway from the two aspects of real-time fluorescence quantitative PCR and western blotting.

Real-time fluorescence quantitative PCR was adopted in detection of PI3K and NF-*κ*B mRNAs expression of cells. Results showed that, after TDH intervention on EC9706 cells, PI3K and NF-*κ*B mRNAs expression showed different degrees of waning; it is significantly different from that in Group BC at 5th and 7th day (*P* < 0.05, Figure 4). Western blotting analysis showed that TDH downregulated proteins expression in PI3K/Akt signaling pathway in transplanted tumor tissues, including PI3K, Akt, p-Akt, and NF-*κ*B. Semiquantitative results indicate that TDH and 5 FU have significant inhibitory effect on proteins expression compared with Group NM (*P* < 0.05, Figure 7 and Table 4); the inhibition of protein expression in PI3K/Akt signaling pathway could be one of mechanisms of the monomer regulating EC cells growth and infiltration. Western blot and PCR results clearly indicated that TDH showed an inhibitory effect on PI3K/Akt-NF-*κ*B transduction cascade in EC cells; specific mechanism involved multiple targets and molecular levels, including RNA transcription and protein expression. But there is another academic question worth studying. Are there some upstream molecules regulated by TDH? As is known to all, the transduction cascade involves signaling pathway existing in both cytoplasm (PI3K/Akt) and nucleus (NF-*κ*B); is it possible for some transmembrane molecules to be involved, which may be upstream proteins, such as epidermal growth factor or its receptor? All of these wait for further research.

Based on the study, it can be concluded that tenacissoside H has explicit antitumor activity on esophageal cancer. Its mechanism has some connection with regulating cell cycle, and decreasing PCNA expression is involved. The intracellular mechanism has relation with mRNA and protein expression in PI3K/Akt-NF-*κ*B transduction cascade.

## Figures and Tables

**Table 1:** mRNAs sequences.

mRNAs	Sequences	Fragments
PI3K		
Fw	5′-GCACCTGAATAGGCAAGTC-3′	172 bp
Rv	5′-TCGCACCACCTCAATAAGT-3′	

NF-*κ*B p65		
Fw	5′-GAGAGCCCTTGCCTCCTTT-3′	106 bp
Rv	5′-CTTCCCTTTGGTCTTTCTG-3′	

*β*-actin		
Fw	5′-TCATCACCATTGGCAATGA-3′	150 bp
Rv	5′-CACTGTGTTGGCGTACAGG-3′	

**Table 2:** Effect of TDH on EC9706 cells transplanted tumor in nude mice ($\overset{-}{X}\pm s$).

Group	Dosage/mg·kg^−1^	Number		Tumor volume/mm^3^		Relative tumor volume/%
		Begin	End	Begin	End	
NM		12	12	258 ± 116	2146 ± 755	12.19 ± 5.10
PCD	20	6	6	254 ± 105	1147 ± 420	6.54 ± 2.44^a^
TDH	100	6	6	260 ± 121	1320 ± 652	7.46 ± 2.67^a^

Data are expressed as mean ± standard error. Data are analyzed by LSD test, ^a^ *P* < 0.05 versus Group BC. Results are considered significant at *P* < 0.05. Three samples are observed in each group. The dosage of drugs is as follows: in Group NM, saline 20 ml·kg^−1^; in Group PCD, 5 FU 20 mg·kg^−1^; in Group TDH, TDH 100 mg·kg^−1^.

**Table 3:** Comparison of PCNA positive cells ratio in tumor tissue of nude mice ($\overset{-}{X}\pm s$, %).

Group	Dosage/mg·kg^−1^	Number		PCNA positive cells ratio/%
		Begin	End	
NM		12	12	95.81 ± 6.32
PCD	20	6	6	48.77 ± 11.56^a^
TDH	100	6	6	53.02 ± 9.95^a^

Note: data were expressed as mean ± standard error of mean. Data were analyzed by LSD test, ^a^ *P* < 0.05 versus Group NM. Results were considered significant at *P* < 0.05. Ten samples were observed in each group.

**Table 4:** Relative gray values of protein ($\overset{-}{X}\pm s$).

Group	PI3K/*β*-actin	Akt/*β*-actin	p-Akt/*β*-actin	NF-*κ*B/*β*-actin
NM	1.184 ± 0.405	1.012 ± 0.223	1.370 ± 0.309	0.559 ± 0.197
PCD	0.411 ± 0.209^a^	0.605 ± 0.193^a^	0.984 ± 0.228^a^	0.327 ± 0.091^a^
TDH	0.528 ± 0.198^a^	0.611 ± 0.217^a^	0.491 ± 0.239^a^	0.405 ± 0.170^a^

Gray values were analyzed with Image J software. Semiquantity on protein bands was conducted. Data were expressed as mean ± standard error. Data were analyzed by LSD test, ^a^ *P* < 0.05 versus Group NM. Results were considered significant at *P* < 0.05. Three samples were observed in each group.
